# *FIP-fve* Stimulates Cell Proliferation and Enhances IL-2 Release by Activating MAP2K3/p38α (MAPK14) Signaling Pathway in Jurkat E6-1 Cells

**DOI:** 10.3389/fnut.2022.881924

**Published:** 2022-05-09

**Authors:** Kefei Gu, Tan Wang, Liying Peng, Yueliang Zhao

**Affiliations:** ^1^Institute for Agri-Food Standards and Testing Technology, Shanghai Academy of Agricultural Sciences, Shanghai, China; ^2^College of Food Science and Technology, Shanghai Ocean University, Shanghai, China; ^3^Laboratory of Quality and Safety Risk Assessment for Aquatic Products on Storage and Preservation (Shanghai), Ministry of Agriculture, Shanghai, China; ^4^Institute of Animal Husbandry and Veterinary Science, Shanghai Academy of Agricultural Science, Shanghai, China

**Keywords:** *FIP-fve*, immunomodulation mechanism, proteomics analysis, MAP2K3/p38α, IL-2

## Abstract

*FIP-fve*, a fungal fruiting body protein from *Flammulina velutipes*, has potential immunomodulatory properties. Here, we investigated the immunomodulation mechanism of *FIP-fve* in Jurkat E6-1 cells by conducting a cell viability assay and IL-2 release assay. Kinase inhibitors experiment and proteomics analysis were also involved in the mechanism study. It was found that *FIP-fve* stimulated cell proliferation and enhanced IL-2 secretion in a dose-dependent manner in Jurkat E6-1 cells. Unbiased high-throughput proteomics analysis showed that 4 T cell immune activation markers, including ZAP-70, CD69, CD82, and KIF23, were upregulated in response to *FIP-fve* treatment. Further pathway analysis indicated that MAP2K3/p38 pathway-related proteins, including MAP2K, p38, ELK, AATF, FOS, and JUN-B, were unregulated. In addition, losmapimod (p38 inhibitor) and gossypetin (MAP2K3 inhibitor) inhibited *FIP-fve* enhanced cell proliferation and IL-2 release in Jurkat E6-1 cells. Our results demonstrate that *FIP-fve* stimulates cell proliferation and enhances IL-2 secretion through MAP2K3/p38α activation.

## Introduction

*FIP-fve* is a fungal fruiting body protein from golden needle mushroom (*Flammulina velutipes*) which is widely consumed in the orient. It has been reported to possess immunomodulatory, antiviral, and antiallergic activities (1–4). The current research showed that *FIP-fve* stimulates mitogenesis of human peripheral blood mononuclear cells (PBMCs), promotes cell proliferation by inducing G_0_/G_1_ to S phase progression, and enhances the transcription of interleukin-2 (IL-2) and interferon-γ (IFN-γ) (5). It was a new mitogen and immunomodulator with therapeutic potential, which may have clinical utility in the adjuvant treatment of diseases, whose mechanisms of action were not fully understood. Therefore it is necessary to further study.

Mitogen-activated protein kinase (MAPK) pathways consisting of the extracellular signal-related kinase (ERK), c-jun amino-terminal kinase (JNK), and p38 mitogen-activated protein kinase signal pathway (p38MAPK/p38) has been reported to play a critical role in the activation of T cells during the immune response (5, 6). p38 is also considered to be involved in positive selection in early thymocyte development (5–12). The upstream signal factors of the p38 signaling pathway include MAP2K3 (MMK3) and MAP2K6 (MKK6). Previous studies demonstrated that T cells activated by *FIP-fve* could secrete IFN-γ and IL-2 and increase the expression of intercellular adhesion molecule-1 (ICAM-1) in a process mediated by the p38 α mitogen-activated protein kinase (p38α) signaling pathway (5). However, it is not clear whether MAP2K3 (MMK3) and MAP2K6 (MKK6) are involved in the immune regulation of T cells by *FIP-fve*.

Janus kinase signal transducer and activator of transcription (JAK/STAT) pathways play a major role in transferring signals from cell-membrane receptors to the nucleus (8). Studies have shown that JAK/STAT pathways are conventionally involved in the MAPK pathway in the regulation of T cells (9, 13–19). However, it is not clear whether JAK/STAT pathway is involved in the immune regulation of T cells induced by *FIP-fve*.

In this study, the Jurkat E6-1 cells, which are activated by exogenous lectin (such as PHA) and can produce a large amount of IL-2, were used to investigate the immunomodulation activity of *FIP-fve*. Cell viability assay, IL-2 release assay, kinase inhibitors, and proteomics analysis were employed to investigate the potential intracellular signal transduction pathways involved in *FIP-fve*-induced Jurkat E6-1 proliferation.

## Materials and Methods

### Chemicals and Reagents

*Flammulina velutipes* was provided by Shanghai Senyuan biology, China. Losmapimod (purity ≥ 98%) was obtained from Shanghai Beyotime Biotechnology (China). Gossypetin (purity ≥ 99%) was purchased from Shanghai ZhenZhun Biotechnology (China). Acetic acid, ammonium sulfate, NaCl, acetonitrile, Tris, and HCl were provided by Sinopharm Chemical Reagent (China). Coomassie Brilliant Blue R250 was obtained from Shanghai yuanye Bio-Technology (China). Cell lysis buffer (N8031) was obtained from Solarbio (China). DMSO, phytohemagglutinin (PHA), protease inhibitor cocktail (P8340), phosphatase inhibitor cocktail 2 (P5726), and phosphatase inhibitor cocktail 3 (Sigma-Aldrich) were obtained from Sigma (USA).

### Isolation and Characterization of *FIP-fve*

*FIP-fve* extraction and purification were performed according to a reported method (1). Briefly, 500 g of the fruiting bodies of *Flammulina velutipes* were homogenized with 500 ml ice-cold 5% acetic acid solution with an ultrasonic processor (FS-N, Shanghai Ultrasonic Instrument, China), followed by sonication for 90 min (600 W, 3 S, 6 S; 90 min in total) in an ice bath. The soluble proteins in the supernatant were precipitated by the addition of ammonium sulfate to 95% saturated. After centrifugation at 2,000 rpm for 5 min, the precipitate was re-suspended in 10 m Mof Tris-HCl buffer (pH 8.0) and dialyzed against 1 L of Tris-HCl buffer at 4°C for 72 h with five times change of dialysis solution (total of 5 L of buffer). After that, the dialysate was applied to a HiTrapQ-HP column (GE, USA), which was previously equilibrated with 10 mM Tris/HCl at pH 8.0. The column was eluted and purified with a gradient of 0–40% 10 mM Tris/HCl at pH 8.0 and 1 M NaCl (on the chromatograph). The active fractions were collected according to the ultraviolet value and further purified on a Superdex75 column (GE, USA). The Superdex 75 column was previously equilibrated with 20 mM Tris/HCl at pH 8.0 containing 150 mM NaCl. Elution was performed with 20 mM Tris-HCl at pH 8.0 containing 150 mM NaCl. The collected fractions were checked by sodium dodecyl sulfate polyacrylamide Gel Electrophoresis (SDS-PAGE) and a BCA assay. Similar fractions were combined. Then a total of 19.3 mg of purified *FIP-fve* was obtained (2.14 mg/ml). The molecular weight of purified *FIP-fve* was measured by matrix-assisted laser desorption ionization time-of-flight mass spectrometry (5800 MALDI-TOF/TOF, AB Sciex, USA) in a positive ion mode, with the calibration ranges of the linear medium molecular weight calibration materials being 5737.609 + 50, 12,362 + 50, and 16,952 + 50 (Sigma, USA).

### SDS/PAGE

The purification of *FIP-fve* was performed according to a previous study (20). Briefly, cellular proteins (15 μg) from each sample were separated by 12% SDS-PAGE (Mini-Protean TGX™ Gels, 10-wellcomb/Bio-Rad). The separation gel was then stained with Coomassie Brilliant Blue R250 for 12 h. After staining, the gel was washed with a destaining solution(10% acetic acid and 25% anhydrous ethanol)until the bands were visualized. Finally, the stained gel was scanned by Image Scanner (GE Healthcare, USA) at a resolution of 300 dpi (full-color mode).

### Cell Culture and Proliferation Assay

Jurkat E6-1 cells were obtained from the American Type Culture Collection (ATCC). The cells were cultured in RPMI 1640 medium (Gibco, Grand Island, USA) supplemented with 10% fetal bovine serum (FBS, Gibco, Grand Island, USA) at 37°C under a 5% CO_2_. Cell viability was examined using the Cell Counting Kit (CCK-8) (Dojindo, Japan) according to previous studies (21–23). Briefly, cells with a density of 1.0 × 10^4^ cells per well were seeded in 96-well microtiter plates overnight. After incubated with vehicle (PBS) or *FIP-fve* at concentrations (25, 50, 100, or 200 μg/ml), the culture medium was discarded to remove the *FIP-fve*. After that, the cells were incubated with 100 μl of CCK-8 solution (10%) in a culture medium for about 1 h. PHA (5 μg/ml) was used as a positive control. The absorbance of the mixture culture medium was measured at 450 nm using a microplate reader (Biotek, USA).

### IL-2 Release Assay

Jurkat E6-1 cells with a density of 1.0 × 10^4^ cells per well were seeded in 96-well microtiter plates overnight. The cells were then treated with vehicle (PBS), *FIP-fve* at various concentrations (25, 50, 100, or 200 μg/ml), or PHA (1.5 or 5.0 μg/ml, positive control) for 6 h. After that, the supernatant was collected, and the amount of IL-2 in the supernatant was measured using an ELISA kit for human IL-2 (USCN Life Science Inc, China). Absorbance was measured at 450 nm with a Bio-Rad 3550 Microplate Reader (Hercules, CA, USA).

### Proteomic Analysis

After being treated with FIP-fve for 24 h, Jurkat E6-1 cells were lysed in cell lysis buffer (Solarbio, China) supplemented with protease inhibitor cocktail (Sigma, USA), phosphatase inhibitor cocktail 2 (Sigma, USA), and phosphatase inhibitor cocktail 3 (Sigma, USA) by ultrasonication on an ice bath for 3 min. The protein concentration was measured using the BCA Protein Assay kit (Shanghai EpiZyme Biotechnology, China). The protein was enzymolyzed and labeled with a TMT reagent according to FASP (24) before being sent to Shanghai Luming Biotechnology Co., Ltd. China for Tandem Mass Tag™ (TMT™) proteomic analysis.

Reverse-phase (RP) separation was performed on an 1100 HPLC System (Agilent, USA) equipped with an Agilent Zorbax Extend RP column (5 μm, 150 × 2.1 mm). The mobile phase was composed of 2% acetonitrile in water (solvent A) and 98% acetonitrile in water (solvent B). The flow rate was 0.3 mL/min in the following gradients: 8 min, 98%A; 8.01 min, 95%A; 28 min, 75%A; 50 min, 60%A; 50.01 min, 10%A; 60 min, 10%A; 60.01 min, 98%A; and 65 min, 98%A. The injection volume was 1 μl. PDA was set at 210 and 280 nm. The samples were harvested from 8 to 50 min, and elution buffer was collected in 10 centrifuge tubes every minute and was numbered. The separated peptides were lyophilized for MS detection.

All analyses were performed by a Q Exactive mass spectrometer (Thermo, USA) equipped with a Nanospray Flex source (Thermo, USA). The samples were loaded and separated by a C-18 column (75 μm × 150 nm, 2 μm, 100 A, Dionex, USA) on an EASYnLC™1200 system (Thermo Fisher Scientific, United States). The flow rate was 300 nl/min, and the linear gradient lasted 90 min (0–55 min, 8%B; 55–79 min, 30%B; 79–80 min, 50% B; 80–90 min, 100% B; mobile phase A = 0.1% FA in water; mobile phase B = 80% ACN/0.1% FA in water). Full MS scans were acquired in the mass range of 300–1,600 m/z with a mass resolution of 70,000, and the AGC target value was set at 1e6. The ten most intense MS peaks were fragmented with higher-energy collisional dissociation (HCD) with an NCE of 32. MS/MS spectra were obtained with a resolution of 17,500 with an AGC target of 2e5 and a max infection time of 8 ms. The Q-E dynamic exclusion was set at 30.0 s and run under positive mode.

Database Search Proteome Discoverer (v 2.2) was used to thoroughly search all of the Q Exactive raw data against the sample protein database. Database searches were performed with trypsin digestion specificity. Cysteine alkylation was considered a fixed modification in the database search. For the protein quantification method, TMT6-plex was selected. The global false discovery rate (FDR) was set to 0.01, and the protein groups considered for quantification required at least 2 peptides.

Xcalibur 2.1 data processing software was used for *FIP-fve* characteristic mass spectrometry identification. Proteome Discoverer TM 2.2 software was used for cell proteomics experimental data analysis, and the UniProt human database was used. The false-positive rate of peptide identification was less than 1%.

### Statistical Analysis

All experimental data were the average of at least three independent experiments. Differences between experimental groups were calculated using the Student’s paired *t*-test. A value of *p* < 0.05 was considered statistically significant.

## Results and Discussion

### Preparation of *FIP-fve*

**FIGURE 1:**
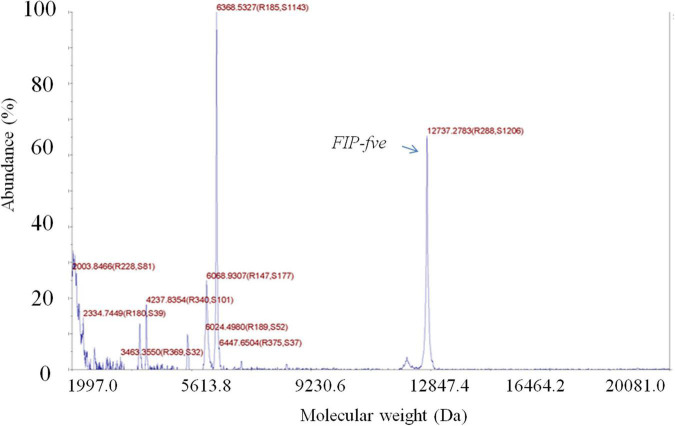
*FIP-fve* is detected by MALDI-TOF/TOF. The molecular weight of FIP-fve was measured by matrix-assisted laser desorption ionization time-of-flight mass spectrometry in a positive ion mode, with the calibration ranges of the linear medium molecular weight calibration materials being 5,737.609 + 50, 12,362 + 50, and 16,952 + 50.

The crude extract yielded two distinct protein bands with apparent molecular masses of 13 and 30 kDa in SDS/PAGE (Supplementary Figure 1A). Then, the crude extract was purified with a HiTrap Q-HP column, and the active *FIP-fve* (13 kDa) (1) obtained from the crude extract was further purified with a Superdex 75 column. The purified *FIP-fve* identified by an LC, yielded a single band with a molecular mass of 13 kDa (Supplementary Figures 1B,C) as determined by the SDS/PAGE assay. A total of 19.3 mg of purified *FIP-fve* with a concentration of 2.14 mg/ml was obtained as determined by the BCA method. Moreover, the molecular weight of the purified *FIP-fve* was 12,737.28 Da as determined by the 5800 MALDI-TOF/TOF (Figure 1).

### *FIP-fve* Stimulates Cell Proliferation and Enhances IL-2 Release

**FIGURE 2:**
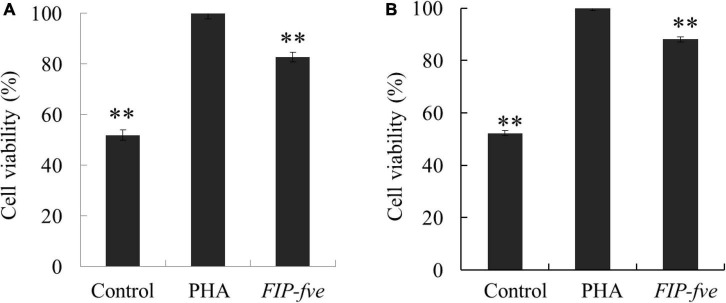
*FIP-fve* treatment results in Jurkat E6-1 cells Proliferation. Jurkat E6-1 cells were treated with *FIP-fve* (100 μg/ml) or PHA (5 μg/ml) for 12 h **(A)** and 24 h **(B)**. Cell viability was assessed by Cell Counting kit-8 assay (CCK-8). PHA was used as a positive control. Values are mean ± SD, *n* = 3, **p < 0.01.

To evaluate the immunomodulation activity of *FIP-fve*, we next determined the effect of *FIP-fve* on cell proliferation and IL-2 release in Jurkat E6-1 cells as the binding of IL-2 to its receptor initiates a series of events that result in cell-cycle progression and T cell proliferation (25). Moreover, the Jurkat E6-1 cells activated by exogenous lectin (such as PHA) can produce a large amount of IL-2. And PHA was used as a positive control (5). The proliferation of Jurkat E6-1 cells was evaluated by CCK-8 kit after 12 and 24 h treatment with control, PHA (5 μg/ml), and *FIP-fve* (100 μg/ml). Although weaker than PHA, *FIP-fve* was found to stimulate Jurkat E6-1 cells proliferation significantly (Figure 2). The results were consistent with a previous study (5) where *FIP-fve* was found to be capable of promoting PBMCs proliferation through enhancing the G_1_ to S phase progression of lymhocytes.

**FIGURE 3:**
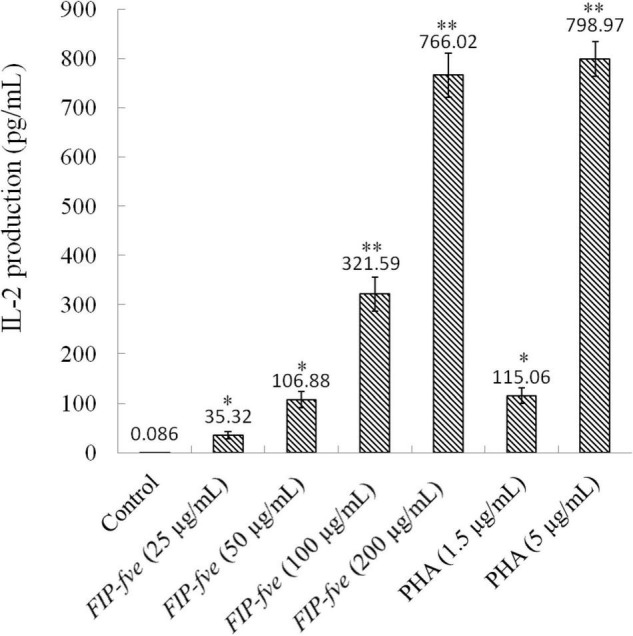
*FIP-fve* induced IL-2 release in Jurkat E6-1 cells. Jurkat E6-1 cells were treated with or without *FIP-fve* (25, 50, 100, and 200 μg/ml) for 6 h. PHA (1.5 and 5 μg/ml) was used as a positive control. IL-2 release was detected by ELISA kit for human IL-2 carried out according to the operating manual of the kit (enzyme marker, optical density, 0). Values are mean ± SD, *n* = 3, *p < 0.05, **p < 0.01.

The *in vitro* immunomodulatory activity of *FIP-fve* was investigated by examining IL-2 release in Jurkat E6-1 cells. Jurkat E6-1 cells were cultured with different concentrations of *FIP-fve* ranging from 35.32 to 766.02 pg/ml for 6 h, and IL-2 production was measured by a human IL-2 ELISA kit. As shown in Figure 3, *FIP-fve* enhanced IL-2 release in a dose-dependent manner. For example, the levels of IL-2 released in the culture medium of cells treated with *FIP-fve* at a concentration of 25, 50, 100, and 200 μg/ml were 35.32, 106.88, 321.59, and 766.02 pg/ml, respectively. Moreover, the levels of IL-2 released from the *FIP-fve* (200 μg/ml) treated cells were almost reachedthat of PHA (5 μg/ml) treated cells (766.02 vs. 798.97 pg/ml). These results indicated that *FIP-fve* could induce T cell immune activation in Jurkat E6-1 cells.

### *FIP-fve* Activated MAP2K3/p38α (MAPK14) Signaling Pathway in Jurkat E6-1 Cells as Evidenced by Proteomics Analysis

**FIGURE 4:**
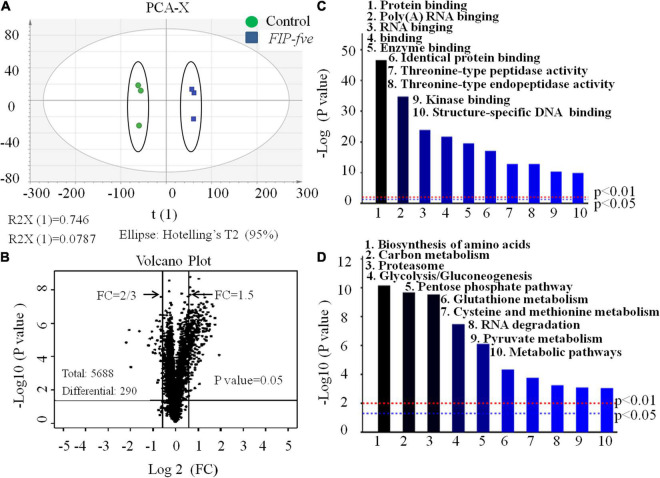
Gene Ontology (GO) molecular function (MF), and Kyoto Encyclopedia of Genes and Genomes (KEGG) analysis of expression of intracellular proteins in *FIP-fve*-stimulated Jurkat E6-1 cells. **(A)** PCA analysis. **(B)** Volcanic map. A black dot in the picture represents a protein. The proteins in the range of FC > 1.5 and FC < 2/3 were differentially expressed proteins. Analysis of GO MF enrichment **(C)** and KEGG **(D)** pathway were conducted by Proteome Discoverer TM 2.2 software based on 290 differential expressed proteins.

The above observations confirmed that *FIP-fve* stimulates cell proliferation and enhances IL-2 release in Jurkat E6-1 cells. Furthermore, unbiased high-throughput proteomics was conducted to explore the mechanism of action of *FIP-fve* in Jurkat E6-1 cells. As shown in Figure 4A, the principal component analysis (PCA) score chart could distinguish the *FIP-fve* group from the control group, indicating that there were differences between the two groups. The samples labeled by enzymatic hydrolysis were analyzed by liquid chromatography-mass spectrometry, and the database was searched. After the blank value was removed, a total of 5,668 credible proteins were detected according to the criteria of score sequence HT > 0 and unique peptide ≥ 1, in which 290 differentially expressed proteins were identified according to FC > 1.5 or FC < 2/3 (fold change, FC) and *p* < 0.05 by the *t*-test (Figure 4B). Based on 290 differentially expressed proteins data, Gene Ontology (GO) molecular function (MF) enrichment and Kyoto Encyclopedia of Genes and Genomes (KEGG) pathway analysis were conducted by Proteome Discoverer TM 2.2 software. As shown in Figure 4C, the main upregulated GO MF were related to protein binding, poly (A) RNA binding, and threonine-type peptidase activity, indicating that *FIP-fve* treatment increased proteins and nucleic acid anabolism in Jurkat E6-1 cells. Moreover, KEGG analysis showed that *FIP-fve* treatment upregulated signal pathways involved in amino acid biosynthesis, carbon metabolism, proteasome, and glycolysis/glycogenesis (Figure 4D). Glycolysis, providing ATP for cell protein and nucleic acid anabolism, is considered to be the main metabolic pathway required for T cell activation. Amino acid synthesis is necessary for protein synthesis, nucleic acid synthesis, regulation of the mtroc1 pathway, and T cell stress pathway. Collectively, GO and KEGG pathway analysis indicated that *FIP-fve* treatment activated pathways related to cell proliferation, further confirming that *FIP-fve* stimulates cell proliferation in Jurkat E6-1 cells.

Among the 290 differentially expressed proteins, 27 proteins were unregulated, including 4 T cell immune activation markers (ZAP-70, CD69, CD82, and KIF23) (10–12, 26–30) (Table 1). In combination with the enhanced IL-2 release, *FIP-fve* was further confirmed to induce T cell immune activation. Moreover, the unregulated 27 proteins include 13 MAPK-pathway-related signaling mediators (Table 1). As shown in Table 1, the expression of MAP2K3 in Jurkat E6-1 cells treated with *FIP-fve* was 1.5 times than that of control. The expression of p38α/MAPK14 was significantly unregulated. The expression of ELK1, AATF, FOS, and JUN-B, which were involved in the nuclear transcription process of the p38 kinase pathway, was also unregulated. Since the MAPK pathway has been reported to be one of the important pathways for T cell activation in the immune response and p38 kinase, the downstream effector of MAPK is considered to be an important kinase for the activation and differentiation of T cells (31), *FIP-fve* might induce immune activation through MAP2K3/p38α (MAPK14) signaling pathway in Jurkat E6-1 cells. In addition, it was also found that the expression of 5 JAK-STAT-pathway related signaling mediators (JAK3, STAT1, STAT3, STAT5B, and STAT6) was unregulated in response to *FIP-fve* (50 μg/ml) treatment (Table 1). As JAK/STAT pathways are conventionally involved in the MAPK pathway in the regulation of T cells, JAK3/STAT signaling pathway may be also involved in the immune regulation activity of *FIP-fve* in Jurkat E6-1 cells.

**TABLE 1:** Upregulated cell activation related proteins in *FIP-fve*-induced Jurkat E6-1 cells.

Signaling pathway	Protein	FC(fold change) ≥ 1.5	*P* ≤ 0.05
			
JAK-STATs	JAK3	↑	
	STAT1		↑
	STAT3		↑
	STAT5B		↑
	STAT6		↑
MAPKs	MAP3K20		↑
	MAP3K2		↑
	MAP2K1		↑
	MAP2K2		↑
	MAP2K3	↑	
	MAP2K7		↑
	MAPK3		↑
	p38α(MAPK14)		↑
	FOS	↑	
	JUN-B	↑	
	ELK		↑
	AATF		↑
	ATF-7		↑
Other cytokines	ICAM-1		↑
	KIF23		↑
	ZAP-70		↑
	CD69	↑	
	CD82		↑
	C9		↑
	GZMA	↑	

### Gossypetin and Losmapimod Attenuated *FIP-fve*-Induced Immune Activation in Jurkat E6-1 Cells

**FIGURE 5:**
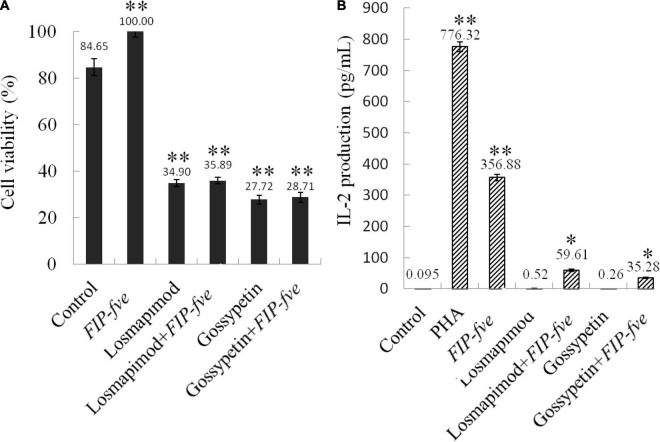
Gossypetin and losmapimod inhibit cell proliferation and IL-2 release in *FIP-fve*-induced Jurkat E6-1 cells. **(A)** Gossypetin and losmapimod attenuated proliferation of *FIP-fve*-induced Jurkat E6-1 cells. Cell viability was determined after treatment within RPMI 1640 medium supplemented with 10% fetal bovine serum *FIP-fve* (100 μg/ml) and/or Losmapimod (0.1 μM/ml) and/or Gossypetin (40 μM/ml) for 12 h. Values are mean ± standard deviation, *n* = 3. PHA (5 μg/ml) was used as a positive control. **(B)** Gossypetin and losmapimod inhibit *FIP-fve*-induced IL-2 release in Jurkat E6-1 cells. IL-2 release was detected by ELISA kit for human IL-2 after treatment with as in **(A)** for 6 h. Values are mean ± SD, *n* = 3, *p < 0.05, **p < 0.01.

Gossypetin (a MAP2K3 inhibitor) and losmapimod (a p38α inhibitor) were then used to study the involvement of the MAP2K3/p38α pathway in *FIP-fve*-induced immune activation in Jurkat E6-1 cells. As shown in Figure 5, 0.1 μM losmapimod and 40 μM gossypetin reduced cells growth stimulated by *FIP-fve* in Jurkat E6-1 cells by 64 and 71%, respectively (Figure 5A). Moreover, losmapimod and gossypetin attenuated *FIP-fve*-enhanced IL-2 released in Jurkat E6-1 cells by 83 and 90%, respectively (Figure 5B). These data in combination with results obtained from proteomics analysis indicate that *FIP-fve* may promote cell proliferation and IL-2 release by activating MAP2K3/p38α signaling pathway in Jurkat E6-1cells.

## Conclusion

*FIP-fve*, a fungal fruiting body protein from *Flammulina velutipes*, stimulated cell proliferation and enhanced IL-2 secretion in Jurkat E6-1 cells. Unbiased high-throughput proteomics analysis indicated that *FIP-fve* may induce immune activation through MAP2K3/p38α (MAPK14) signaling pathway in Jurkat E6-1 cells. Gossypetin (a MAP2K3 inhibitor) and losmapimod (a p38α inhibitor) attenuated cells growth stimulated by *FIP-fve* and IL-2 release enhanced by *FIP-fve*, further confirming that *FIP-fve* stimulates cell proliferation and enhances IL-2 secretion through MAP2K3/p38α activation in Jurkat E6-1 cells.

## Data Availability Statement

The mass spectrometry proteomics data have been deposited to the ProteomeXchange Consortium via the PRIDE [1] partner repository with the dataset identifier PXD032302.

## Author Contributions

KG and YZ conceived the study. KG, TW, and YZ contributed to the design and drafted the work. KG, TW, and LP collected the data. KG and TW analyzed the data. All authors interpreted the data, read the manuscript, and approved the final version.

## Conflict of Interest

The authors declare that the research was conducted in the absence of any commercial or financial relationships that could be construed as a potential conflict of interest.

## Publisher’s Note

All claims expressed in this article are solely those of the authors and do not necessarily represent those of their affiliated organizations, or those of the publisher, the editors and the reviewers. Any product that may be evaluated in this article, or claim that may be made by its manufacturer, is not guaranteed or endorsed by the publisher.
