# Supplementary material for: FIP-fve Stimulates Cell Proliferation and Enhances IL-2 Release by Activating MAP2K3/p38α (MAPK14) Signaling Pathway in Jurkat E6-1 Cells
Source: Front Nutr. 2022 May 9;9:881924. doi: 10.3389/fnut.2022.881924 (PMC9125247; doi:10.3389/fnut.2022.881924)
Supplement: Supplementary file 1 [file Table_1.docx]

**Supplementary Materials For:**

FIP-fve stimulates cell proliferation and enhances IL-2 release by activating MAP2K3/p38α（MAPK14）signaling pathway in Jurkat E6-1 cells

Kefei Gu^1,#^, Tan Wang^1,2,3,#^, Liying Peng^4^, Yueliang Zhao^2,3,*^

^1^ Institute for Agri-food Standards & Testing Technology, Shanghai Academy of Agricultural Sciences, Shanghai, China

^2^ College of Food Science and Technology, Shanghai Ocean University, Shanghai, 201306, China

^3^ Laboratory of Quality and Safety Risk Assessment for Aquatic Products on Storage and Preservation (Shanghai), Ministry of Agriculture, Shanghai, China

^4^ Institute of Animal Husbandry & Veterinary Science, Shanghai Academy of Agricultural Science, Shanghai, China

To whom correspondence should be addressed:

*Dr. Yueliang Zhao, College of Food Science and Technology, Shanghai Ocean University, E-mail: [yueliang2110@163.com](mailto:yueliang2110@163.com), ylzhao@shou.edu.cn





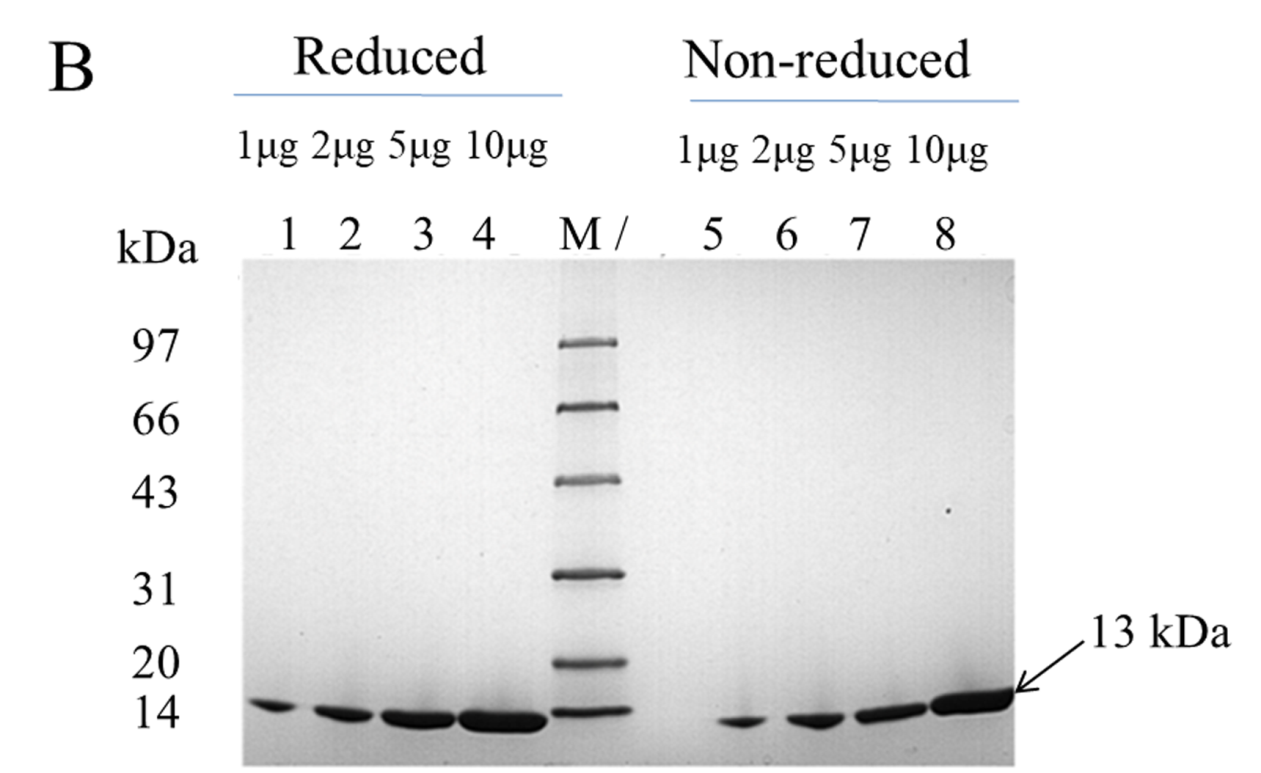


**
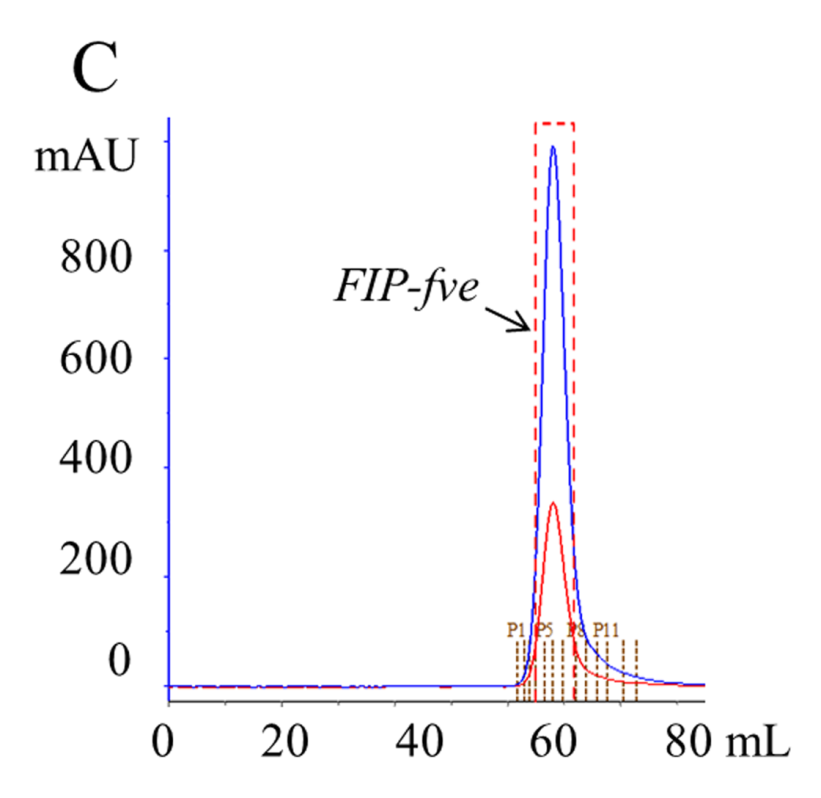
**

**Image 1_v1**. Purification and analysis of FIP-fve. (A) crude extract of *FIP-fve* was detected by reduced/nonreduced 15% SDS-PAGE. Lane 1 crude extract of *FIP-fve*. Lane M, protein marker (phosphorylase B (97 kDa), bovine albumin (66 kDa), ovalbumin (43 kDa), carbonic anhydrase (31 kDa), trypsin inhibitor (20 kDa), lactalbumin (14 kDa)).
